# Supplementary material for: A Paradoxical Vasodilatory Nutraceutical Intervention for Prevention and Attenuation of Migraine—A Hypothetical Review
Source: Nutrients. 2020 Aug 18;12(8):2487. doi: 10.3390/nu12082487 (PMC7468811; doi:10.3390/nu12082487)
Supplement: Supplementary file 1 [file nutrients-12-02487-s001.pdf]

nutrients MDPI

Supplementary Files

Table S1. Animal studies of L-arginine on vascular tone.

Study	*Animal*	*Dose*	*Mode of Administration*	*Treatment Frequency*	*Treatment Duration*	*Medical Condition*	*Effect on Serum Measures (unless otherwise stated)*
Javanmard 2009 [73]	Rabbit	3%	P.o.	Dietary	1 month	Hypercholesterolaemia	Increased eNOS expression intensity in aorta
Howell 2009 [63]	Rat	95 mM	P.o.	Dietary	2 weeks	Hypoxia	Maintained pulmonary arterial pressure, inhibited increased precapillary resistance, maintained decreased vascular resistance
Kharazmi 2015 [51]	Rat	0.01 M	Postmortem vascular infusion	Single	8 weeks	Diabetes	Increased GABA-induced vasodilatation in both diabetic and non-diabetic animals
Mendrinos 2010 [52]	Minipig	30 µl 1 mM	Intravitreal	Single	30 mins.	Retinal vein occlusion	Persistently increased arteriolar diameter
Morita 2014 [72]	Rat, rabbit	2.85 mM/kg	P.o.	Single	4 hours	None	Increased blood NOx (nitrite + nitrate) and cGMP
Ou 2010 [74]	Rat	500 mg/kg	I.p.	Once daily	3, 5 weeks	Pulmonary hypertension	Decreased pulmonary artery wall thickness. Increased NO production, eNOS expression and phosphorylation
Saleh 2011 [105]	Rabbit	2.25%	P.o.	Dietary	28 days	Hypercholesterolaemia	Prevented aortic intimal thickening
Schreiber 2017 [62]	Rat	300 mg/kg	P.o.	Once daily	14 days	Pulmonary hypertension	Decreased pulmonary artery pressure, increased vascular elasticity, palliated small vessel disease
Feng 2013 [61]	Rat	3%	P.o.	Dietary	4 weeks	Insulin-resistance hypertension	Reversed vessel thickness, increased NO and cGMP back to non-hypertensive controls, decreased systolic blood pressure

Study first authors and years are listed with animals used, doses and modes of administration, treatment frequencies and durations, induced medical conditions of the subjects, and drug effects on serum measures. P.o. = per os; i.p. = intraperitoneal; eNOS = endothelial nitric-oxide synthase; GABA = gamma aminobutyric acid; cGMP = cyclic guanosine monophosphate; NO = nitric oxide.

Table S2. Human studies of L-arginine on vascular tone.

Study	*Registration No.*	*Dose (g)* p.o.	*Treatment Frequency*	*Treatment Duration*	*Medical Condition*	*Effect on Serum Measures (unless otherwise stated)*	*Adverse or Side Effects*
Ast 2010 [69]	NA	2, 4	Thrice daily	4 weeks	Primary hypertension	Decreased blood pressure with 12g daily (4g x3)	None reported
Camarena Pulido 2016 [67]	NCT 02363348	3	Once daily	20 weeks	Risk of pre-eclampsia	Decreased cases of pre-eclampsia	Mild dyspepsia
Chen 2010 [80]	NA	5.2	Once daily	3 weeks	Old age	Increased anaerobic threshold	None
Deveaux 2016 [53]	NCT02354794	1.5	Thrice daily	4 weeks	Overweight, cardiometabolic risk	In those with lower baseline L-arginine, attenuated decrease in flow-mediated dilatation	None
Fahs 2009 [55]	NA	7	4 times, before exercise, 72 hours apart	16 days	None	After exercise: Decreased brachial stiffness. Increased central aortic stiffness, reactive hyperaemia, forearm blood flow	None reported
Jahangir 2009 [106]	NA	9	Once daily	4 days	Coronary artery disease	Increased homocysteine:methionine ratio	None reported
Lucotti 2009 [58]	NCT00408577	6.4	Once daily	6 months	Cardiovascular disease, after aortocoronary bypass	Decreased ADMA. Increased cGMP, L-arginine:ADMA ratio, reactive hyperaemia	None
Morris 2013 [77]	NCT01796678	0.1/kg	Thrice daily	5 days	Sickle cell disease	Decreased vaso-occlusive pain score	None
Neri 2010 [70]	NCT00974714	4	Once daily	12 weeks	Pregnant, chronic mild hypertension	No change in blood pressure, but fewer received anti-hypertensives	None reported
Orozco-Gutiérrez 2010 [50]	NA	4	Twice daily	2 months	Heart failure	Decreased pulmonary artery, diastolic and systolic artery pressures. Increased duration on treadmill	Gastrointestinal distress
Pahlavani 2017 [49]	IRCT2013121515807N1	2	Once daily	45 days	None	Increased maximal oxygen uptake	Skin dermatitis, stomach problems
Siasos 2009 [54]	NA	7	Thrice daily	3 days	Smoking	Increased flow-mediated dilatation. Decreased carotid-femoral pulse wave velocity	None reported
Vadillo-Ortega 2011 [68]	NCT00469846	3.3	Twice daily	19 weeks	Risk of pre-eclampsia	With antioxidant vitamins: Decreased pre-eclampsia incidence	Nausea, dyspepsia, dizziness, palpitations, headache

Study first authors and years are listed with clinical trial registration numbers, oral doses in grams, treatment frequencies and durations, existing medical conditions of the subjects, drug effects on serum measures and adverse/side effects reported. “None reported” = study did not mention adverse/side effects; “none” = there were no adverse/side effects from the treatment; p.o. = per os; NA = not available.

Table S3. Animal studies of AGE on vascular tone.

Study	*Animal*	*Dose*	*Mode of Administration*	*Treatment Frequency*	*Treatment Duration*	*Medical Condition*	*Effect on Serum Measures (unless otherwise stated)*
Aguilera 2010 [86]	Rat	1.2 ml/kg	I.p.	30 mins. before, at onset of, or 1 hr. after reperfusion	2 hrs. infarct, 4 hrs. reperfusion	Middle cerebral artery occlusion causing focal ischemia	Onset of reperfusion: Decreased 2-hr. infarct area
Cemil 2016 [107]	Rat	250 mg/kg	P.o.	Once daily	15 days	CNS injury	CNS tissue: Decreased ischaemia
Colin-Gonzalez 2011 [87]	Rat	1.2 ml/kg	I.p.	Single (reperfusion onset)	1 hour infarct, 24 hours reperfusion	Middle cerebral artery occlusion causing focal ischemia	After 1 hr. ischaemia, 24 hrs. reperfusion: Decreased infarct area
Perez-Torres 2016 [85]	Rat	125 mg/kg	I.a.	Twice daily	1 month	Metabolic syndrome	Decreased systolic blood pressure and coronary vascular resistance. Increased vascular function, heart NO, citrulline, NOx

Study first authors and years are listed with animals used, doses and modes of administration, treatment frequencies and durations, induced medical conditions of the subjects, and drug effects on serum measures. I.p. = intraperitoneal; p.o. = per os; i.a. = intra-abdominal; CNS = central nervous system.

Table S4. Human studies of AGE on vascular tone. .

Study	*Registration No.*	*Dose (g)* **p.o.**	*Treatment Frequency*	*Treatment Duration*	*Medical Condition*	*Effect on Serum Measures (unless otherwise stated)*	*Adverse or Side Effects*
Ahmadi 2013 [90]	NA	0.25	Once daily	1 year	Medium cardiovascular risk	Decreased homocysteine. Increased temperature rebound	None reported
Budoff 2009 [83]	NCT01534910	0.25	Once daily	1 year	Medium cardiovascular risk	With supplements including 0.1g L-arginine: Decreased homocysteine. Increased temperature rebound	None reported
Larijani 2013 [91]	NCT00860847	0.3	Every 3 months	1 year	None	With 30 mg CoQ10: Improved pulse-wave velocity and digital thermal monitoring	None reported
Ried 2010 [92]	ACTRN12609000151235	0.96	Once daily	12 weeks	Systolic hypertension	Containing 2.4 mg S-allyl-cysteine: In hypertensive patients, systolic blood pressure decreased. In normotensive patients, no change	Belching, reflux, taste sensations
Ried 2013 [94]	ACTRN12611000581965	0.24, 0.48, 0.96	Once daily	12 weeks	Systolic hypertension	Containing 0.6, 1.2, 2.4 mg S-allyl-cysteine: Decreased systolic blood pressure in patients taking 0.48g AGE (1.2g S-allyl-cysteine)	Constipation, bloating, flatulence, reflux, garlic taste, difficulty swallowing the capsules, dry mouth, cough
Ried 2016 [48]	ACTRN12613000747729	1.2	Once daily	12 weeks	Hypertension	Containing 1.2 mg S-allyl-cysteine: Improved central blood pressure, central pulse pressure, mean arterial pressure, augmentation pressure, pulse-wave velocity, arterial stiffness	Reflux, burping, bloating. No increased bleeding risk in patients on blood-thinning drugs. Improved digestion
Reid 2018 [93]	ACTRN12616000185460	1.2	Once daily	12 weeks	Hypertension	Containing 1.2 mg S-allyl-cysteine: Decreased mean, central and pulse blood pressures, arterial stiffness	Garlic taste, burping
Seo 2012 [108]	NA	0.08	Once daily	12 weeks	Postmenopausal	Decreased homocysteine	None reported

Study first authors and years are listed with clinical trial registration numbers, oral doses in grams, treatment frequencies and durations, existing medical conditions of the subjects, drug effects on serum measures and adverse/side effects reported. “None reported” = study did not mention adverse/side effects; “none” = there were no adverse/side effects from the treatment; NA = not available.

Table S5. Currently commonly used approaches to treat migraines.

Migraine Treatment	Examples	Considerations
Acute medication	Analgesics [34, 35, 37, 109, 110]<br><br>Antiemetics [31, 37, 111, 112]<br><br>Barbiturates [35, 109, 110, 113, 114]<br><br>Bolutinum toxin and muscle relaxants [32, 109, 112, 114, 115]<br><br>CGRP receptor antagonists (gepants) [116]<br><br>Ergot derivatives [34, 36, 37, 110-112, 114]<br><br>Local anaesthetics [37]<br><br>NSAIDs [32, 35, 37, 109, 110, 112, 115, 117]<br><br>Opioids/opiates [31, 34, 35, 109-111]<br><br>Selective serotonin reuptake inhibitors (SSRIs) [36, 117, 118]<br><br>Triptans [34-37, 110-112, 114, 116, 119]	Can induce migraine chronicity/recurrence, contraindicated in pregnancy, adverse effects, tolerance and dependence, serotonin interaction effects, require multiple doses, expensive, contraindicated in valvular disorders, short-term efficacy, early migraine recurrence, contraindicated in bleeding disorders, contraindicated in asthma, contraindicated in urticaria, contraindicated in renal impairment, contraindicated in peptic ulcers or recent gastrointestinal bleeding, abuse, rebound headache, additional dietary and management requirements, contraindications with MAOIs or SSRIs, contraindicated in cardiovascular disorders, incomplete and inconsistent pain relief, inefficacy during aura/premonitory phase [34-37, 110, 111, 113-116, 120-123]
Prophylactic medication	Alpha-2 agonists [112, 113]<br><br>Angiotensin antihypertensives [112, 114, 124]<br><br>Anticonvulsants or antiepileptics [32, 36, 112, 114, 117, 124]<br><br>Beta blockers [36, 112, 114, 117, 124]<br><br>Bolutinum toxin and muscle relaxants [32, 109, 112, 114, 115]<br><br>Calcium channel blockers [36, 114, 117, 125]<br><br>Monoamine oxidase inhibitors (MAOIs) [36]<br><br>NMDA receptor antagonists [115, 126]<br><br>NSAIDs [32, 35, 37, 109, 110, 112, 115, 117]<br><br>Parenteral nonsteroidal agents and corticosteroids [37, 111, 112, 127]<br><br>Selective norepinephrine reuptake	Adverse effects, ineffective at low doses, contraindicated in pregnancy, expensive, contraindicated in diabetes, long time to take effect, contraindicated in myocardial conduction blockages, drug interactions, contraindicated in bleeding disorders, contraindicated in asthma, contraindicated in urticaria, contraindicated in renal impairment contraindicated in peptic ulcers or recent gastrointestinal bleeding, additional dietary and management requirements, contraindications with MAOIs, contraindicated in glaucoma, anticholinergic effects, contraindicated in epilepsy, contraindicated in acute heart-attack recovery [34-37, 111-115, 126-132]

	inhibitors (SNRIs) [114]<br><br>Selective serotonin reuptake inhibitors (SSRIs) [36, 117, 118]<br><br>Serotonin antagonists [112, 113, 117]<br><br>Tricyclic antidepressants [32, 36, 109, 112, 114, 115, 117, 124, 128]	
Acupuncture [133-141]	N/A	Insufficient or conflicting evidence, ineffective, small effect size [133-135, 137, 138, 140]
Meditation [142-146]	Distraction technique [145]<br><br>Externally Focused Secular Meditation [142]<br><br>Internally Focused Secular Meditation [142]<br><br>Mindfulness [144, 146]<br><br>Progressive Muscle Relaxation [142]<br><br>Spiritual Meditation [142]	No effect on pain sensitivity, ineffective, insufficient evidence, distraction insufficient, meditation practice takes time [142-146]

Each treatment approach is listed alongside examples and their caveats. NSAID = non-steroidal anti-inflammatory drug; CGRP = calcitonin gene-related peptide.
